# Evaluation of Anti-*Candida albicans* Activities of Herbal Preparations Sold at the Kumasi Central Market in the Ashanti Region of Ghana

**DOI:** 10.1155/2023/6162532

**Published:** 2023-04-11

**Authors:** Seth A. Domfeh, Godfred Kyeremeh, Mark Belifini

**Affiliations:** ^1^Department of Biochemistry and Biotechnology, Faculty of Biosciences, Kwame Nkrumah University of Science and Technology, Kumasi, Ghana; ^2^Department of Medical Laboratory Technology, Faculty of Health Sciences, Garden City University College, Kenyasi-Kumasi, Ghana

## Abstract

*Candida albicans* (*C. albicans*) is predominantly the leading cause of candidiasis among women with urogenital candidiasis. Since most people in resource-limited countries depend on herbal medicine for their primary care needs, many herbal drugs are sold to manage various infectious diseases. This study, therefore, evaluated the anti-*C. albicans* activities of five selected herbal preparations indicated for treating candidiasis sold at the Kumasi Central Market in the Ashanti Region of Ghana. The market was divided into five clusters, and one herbal preparation was randomly selected from each cluster. Using the Kirby Bauer disc diffusion antimicrobial susceptibility test, the herbal preparations were tested against clinically isolated *C. albicans*. Fluconazole, a standard antifungal drug, was included in the evaluation as a positive control. The experiments were performed on three different days and each in triplicates. Among the five selected herbal preparations, only one was effective against *C. albicans* with a mean inhibition zone of 19.1 mm. This effective herbal drug was prepared from *Centella asiatica* sap, *Turnera microphylla* leaves, and *Vitex agnus-castus* leaves. The results suggest that not all the herbal preparations selected were effective against *C. albicans*. Hence, we recommend that the authorities continually check the effectiveness of the herbal preparations on the market.

## 1. Background

Candidiasis is a fungal disease caused by *Candida* spp., mostly *Candida albicans* (*C. albicans*) accounting for about 60% of the reported cases [1]. Other non*-albicans*, including *Candida glabrata*, *Candida parapsilosis*, *Candida krusei*, *Candida tropicalis*, *Candida vaginitis,* and *Candida auris*, have also been identified to cause candidiasis [2, 3]. Candidiasis spans from mucocutaneous mycoses such as urogenital and oral candidiasis to life-threatening invasive forms such as candidaemia and hepatosplenic candidiasis [4]. In Ghana, most studies have focused on urogenital candidiasis, with a prevalence of 21–25.5% [5, 6]. As a result, different herbal preparations are indicated for treating urogenital candidiasis on the market.

Plants have been used in traditional herbal medicine for years [7, 8]. In some parts of the world, plants and herbs are still the primary sources of remedies used in treating diseases [9]. For example, *Centella asiatica* is used in managing varied conditions including, but not limited to, diseases of the female urogenital tract, fever, psoriasis, and wounds [10]. Also, in low- and middle-income countries (LMICs), about 80% of people depend on medicinal plants for their primary healthcare needs [11]. Moreover, these medicinal plants are easily accessible in these countries [12]. Although some herbal preparations on the market are effective, not much can be said about others because there is limited postmarket surveillance. Also, some people are into preparing herbal drugs because of the proceeds and not the effectiveness of their preparations [13].

Herbal preparations on the market are generally safe but can cause severe adverse risks among consumers [14]. Also, the effectiveness of these herbal preparations cannot always be guaranteed [15, 16]. In Ghana, efforts are being made by the Food and Drugs Authority to assess the safety and efficacy of herbal preparations before they are introduced into the market. However, some herbal preparations still get to the market without safety and efficacy evaluation. Also, there is limited postmarket safety and efficacy surveillance on herbal preparations, thereby subjecting consumers to ineffective treatment of their disease conditions. A study in Kumasi in the Ashanti Region of Ghana using a qualitative semistructured interview reported that some herbal preparations on the market are ineffective, according to responders [16]. Also, there are limited studies on validating antimicrobial herbal preparations on the markets in Ghana.

Over the years, herbal preparations have been used in managing yeast and bacterial infections due to their effectiveness and low cost [17]. A previous study has shown that two medicinal plants, *Lawsonia inermis* and *Portulaca oleracea* have significant anti-*Candida albicans* (anti-*C. albicans*) activities [18]. Also, several plant extracts have been reported to have anti-*C. albicans* activities, including *Allium sativum*, *Cinnamomum verum,* and *Origanum vulgare* [19–23]. Moreover, extracts from *Alchornea cordifolia* (*A. cordifolia*), *Spathodea campanulata* (*S. campanulata*), and *Afzelia africana* (*A. africana*) have been reported to inhibit the growth of *C. albicans* [24–26]. These studies assessed individual medicinal plants without the combinations of the plant extracts. However, one fascinating thing about herbal medicine products is that most herbal formulations on the market are polyherbal. Also, there are limited studies on the postmarket efficacy of most polyherbal formulations. Hence, this study was imperative to evaluate the anti-*C. albicans* activities of herbal preparations indicated for treating candidiasis sold at the Kumasi Central Market in the Ashanti region of Ghana. Thus, this study targets a real-life concern of evaluating herbal preparations on the market for managing candidiasis.

## 2. Materials and Methods

### 2.1. Study Design and Study Site

A cross-sectional study was conducted from March to May 2022 to evaluate the anti-*C. albicans* activities of five herbal preparations sold at the Kumasi Central Market in the Ashanti Region of Ghana. The laboratory evaluations were conducted at the Department of Medical Laboratory Technology, Garden City University College, Kenyasi-Kumasi, Ghana.

### 2.2. Selection of Herbal Preparations

The Kumasi Central Market was divided into five clusters, and one herbal preparation with an indication for treating candidiasis was randomly selected from each cluster. The herbal preparations were given coded names due to ethical considerations (*A*, *B*, *C*, *D,* and *E*).

### 2.3. Confirmation of Clinically Isolated *Candida albicans*

Clinically isolated *C. albicans* were obtained from the Microbiology Laboratory of the Komfo Anokye Teaching Hospital in Kumasi, Ghana. The *C. albicans* were inoculated on the sabouraud dextrose agar (SDA) and incubated for 24 hours at 37°C. The isolates were confirmed using the Integral System Yeasts Plus (Liofilchem, Italy), a system for identifying clinically important yeasts, by following the manufacturer's instructions [27]. Also, the confirmation was supported by performing a germ tube test on the *C. albicans* isolates [28].

### 2.4. Anti-*Candida albicans* Susceptibility Testing

Using the Kirby Bauer disc diffusion antimicrobial susceptibility test, the herbal preparations were tested against the clinically isolated *C. albicans*. A sterile filter paper was cut into about 6 mm and impregnated with the herbal preparations by allowing the paper to absorb the preparations until it could not absorb any more and allowed to dry as previously described [17]. Mueller Hinton agar plates were inoculated with 0.5 McFarland *C. albicans* suspension and dried for 5 minutes. Each herbal preparation was assigned a plate with fluconazole as a positive control in the experiment. The impregnated discs were placed on the plates and incubated for 24 hours at 37°C. A filter paper impregnated with sterile water was included as a negative control. The zone of inhibition was assessed as previously described [17]. The experiments were performed on three separate occasions and each in triplicates.

### 2.5. Data Analysis

Data were analysed using the Statistical Package for Social Sciences Statistical Software (version 20.0, IBM Corporation, USA). The data were expressed in means and standard deviations. Where appropriate, the differences in the means of inhibition zones were assessed using a student's *t*-test or two-way ANOVA (analysis of variance). The statistical significance was accepted in all comparisons at a *p* value of less than 0.05.

## 3. Results

### 3.1. Constituents of the Selected Herbal Preparations

From the five randomly selected herbal preparations at the Kumasi Central Market in the Ashanti Region of Ghana, *A. cordifolia* was the most common plant constituent. Also, the herbal preparations were constituted using at least two medicinal plants (Table 1).

### 3.2. Anti-*C. albicans* Activities of the Selected Herbal Preparations

**Figure 1:**
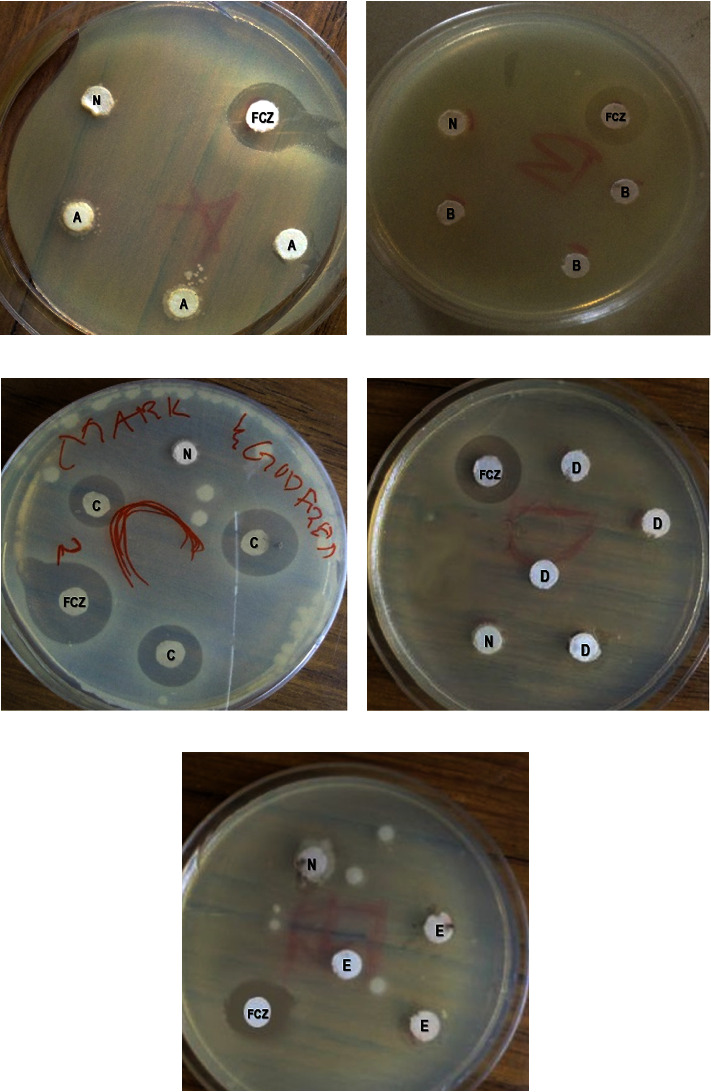
Inhibition of *C. albicans* growth by herbal preparations. The pictures are representatives of three different experiments. FCZ: fluconazole; N: negative control; (a–e): herbal preparations (A–E).

Among the five selected herbal preparations indicated for treating candidiasis or yeast infection sold at the market, only the herbal preparation *C* inhibited the growth of *C. albicans* (Figure 1(c), plate C). Intriguingly, the impregnated discs used for the herbal preparation C did not affect the zone of inhibition (*p*=0.6553). Also, the different days on which the tests were performed did not affect the zone of inhibition (*p*=0.1254), as shown in Table 2.

### 3.3. Comparison of Inhibition Zones of the Herbal Preparation C and Fluconazole

**Figure 2:**
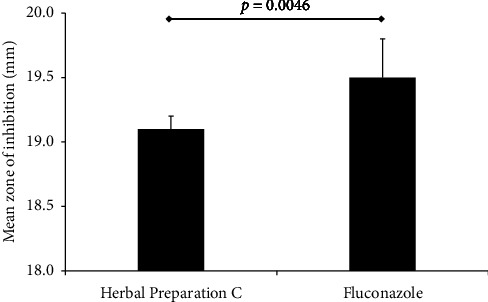
Comparison of inhibition zones of the herbal preparation C and fluconazole. The statistical difference was assessed using a student's *t-*test.

The inhibition zone of fluconazole (19.5 ± 0.3) was statistically higher (*p*=0.0046) than that of the inhibition zone of the herbal preparation C (19.1 ± 0.1), as shown in Figure 2.

## 4. Discussion

In Ghana, most herbal medicine practitioners combine various plant parts or species to make herbal mixtures. This combination is due to these medicinal plants' additive or synergistic effects. Over-the-counter herbal preparations are popular, with an estimated 12% of the world population accessing herbal medicine. Also, in resource-limited countries, over 80% rely on medicinal plants for their primary healthcare needs, resulting in an estimated world market of 50 billion USD in the commercial herbal medicine industry, with an annual growth of 6.5% [11, 29]. Although herbal supplements are popular worldwide, most herbal preparations on the market have unreliable efficacy and low quality. Hence, clinicians and scientists are usually concerned about the safety, effectiveness, and consistency of these herbal mixtures (reviewed in [30]).

Among the five herbal preparations selected for the study from the market, only one of them inhibited the growth of *C. albicans* with an appreciable zone of inhibition (19.1 mm). This effective herbal mixture was prepared from *Centella asiatica* (*C. asiatica*), *Turnera microphylla* (*T. microphylla*) and *Vitex agnus-castus* (*V. agnus-castus*). Hence, the anti-*C. albicans* activity of this herbal preparation could be an additive or synergistic effect between the active ingredients of the medicinal plants because *C. asiatica* and *V. agnus-castus* have been reported to exhibit anti-*C. albicans* activities [31, 32]. However, there is limited data on the anti-*C. albicans* activity of *T. microphylla*; hence, further studies are required to assess this plant to know its contributing effect in this effective herbal preparation for managing candidiasis.

Extracts from *C. asiatica* have been used in ethnomedicine for managing lupus, eczema, psoriasis, wounds, and diseases of the female urogenital tract [10, 33]. Also, 100 g of fresh *C. asiatica* delivers 13.8 mg of vitamin C (reviewed in [34]). Vitamin C activates the body's immune system by stimulating leukocyte activities [35]. Hence, these medicinal and nutritional benefits of *C. asiatica* support the anti-*C. albicans* activity of the herbal preparation. Ethnomedically, *V. agnus-castus* extracts are used to manage menopausal problems and menstrual disorders. Also, this medicinal plant has other pharmacological effects, including antifungal, antibacterial, and anti-inflammatory effects (reviewed in [36]). These reported pharmacological effects support the inclusion of *V. agnus-castus* in the herbal preparation with effective anti-*C. albicans* activity.

There are several mechanisms through which agents exhibit their antifungal effects. For example, fluconazole, like the other azoles, involves disruption of the conversion of lanosterol to ergosterol and subsequent disruption of fungal membranes. Also, nystatin binds to sterols in the plasma membranes of fungi leading to fungal cell death. *C. asiatica* contains pentacyclic triterpenes including, but not limited to, asiaticoside, madecassic acid, and asiatic acid [37]. Asiatic acid has various therapeutic properties, including antifungal activity against *C. albicans*. Drug efflux overexpression is one of the most common mechanisms of drug resistance in *C. albicans* (reviewed in [38]). Interestingly, asiatic acid has been shown to inhibit efflux pump activity and morphological transformation in *C. albicans* [39]. Hence, it could be concluded that the inhibition of efflux pump activity and morphological transformation by asiatic acid could be one of the molecular mechanisms via which the effective herbal preparation inhibits *C. albicans* growth.

Moreover, *V. agnus-castus* contains essential oils, which have been reported to inhibit the growth of *C. albicans* similar to amphotericin [40]. Farnesyl pyrophosphate synthase is a vital enzyme in *Candida* spp. required for ergosterol biosynthesis, the main component in the cell membrane [41–43]. Hence, targeting this enzyme is suicidal for *Candida* spp., and essential oils from *V. agnus-castus* (caryophyllene and verticiol) have good binding affinities to this enzyme. Caryophyllene binds to the enzyme's active site via hydrophobic interactions, whereas verticiol forms a hydrogen bond with the Asp156 in the enzyme's active site, justifying the anticandidal activity of *V. agnus-castus* [40]. Also, the binding activities of caryophyllene and verticiol could be another molecular mechanism via which the effective herbal preparation inhibits *C. albicans* growth.

The active herbal preparation against *C. albicans* exhibited an inhibition zone of 19.1 mm. Compared to the standard drug, fluconazole, there was a statistical difference in the inhibition zone, with fluconazole having a higher zone of inhibition (19.5 mm). Even though there is no standard interpretation for the zone of inhibition for the active herbal preparation, it could be accepted to be effective for treating candidiasis since it has a zone of inhibition of ≥19 mm. However, a previous study that assessed the antifungal activity of *Flos rosae chinensis* on *C. albicans* found that this medicinal plant was more effective than fluconazole in inhibiting the growth of *C. albicans* with an inhibition zone of 22 mm compared to fluconazole (18 mm) [44]. Although this current study reported an antifungal activity of a polyherbal, it still supports the effectiveness of herbal medicines for managing infectious diseases, including candidiasis.

Plant constituents of some of the selected herbal preparations, such as *A. cordifolia*, *S. campanulata*, and *A. africana*, have been reported to inhibit the growth of *C. albicans* [24–26]. However, the polyherbal mixture containing these plant extracts could not inhibit the growth of *C. albicans*. This low efficacy could be attributed to inconsistent processing methods used by manufacturers and different therapeutic potentials in herbal preparations resulting from the age, harvesting time, geographical location, and postharvest handling of the plants. Moreover, the combination of these plants could hamper their antimicrobial activities via antagonism [45].

## 5. Conclusions and Recommendations

Only one of the five selected herbal preparations indicated for treating candidiasis was effective against the growth of *C. albicans*. The effective herbal preparation was prepared from *C. asiatica*, *T. microphylla,* and *V. agnus-castus*. Previous studies have shown that active agents from *C. asiatica* and *V. agnus-castus* inhibit *C. albicans* growth by inhibiting efflux pump and farnesyl pyrophosphate synthase activities [39, 40], supporting the anti-*C. albicans* activity of the effective herbal preparation. This study has revealed that not all herbal preparations on the market are effective. Hence, authorities should continually check the effectiveness of herbal preparations on the market. Further, persons should use herbal preparations prescribed by authorised herbal medicine practitioners. We also recommend similar studies in different markets in Ghana using larger sample sizes.

## 6. Limitations of the Study

The study was performed using a small sample size due to financial constraints, which could account for the low effective rate of the herbal preparations. Nonetheless, our findings could serve as the baseline data for further studies on commercially processed herbal preparations.

## Figures and Tables

**Table 1:** Plant constituents and indications of the selected herbal preparations.

Preparations	Plant constituents	Indications
*A*	*Alchornea cordifolia* leaves *Spathodea campanulata* leaves	Gonorrhoea, candidiasis, and other STDs in men and women

*B*	*Citrus aurantifolia* fruits *Vismia guineensis* leaves *Afzelia africana* stem bark	Candidiasis, painful menstruation, and proper vagina care

*C*	*Centella asiatica* sap *Turnera microphylla* leaves *Vitex agnus-castus* leaves	*Staphylococcus* spp, yeast infection, and toilet infection

*D*	*Alchornea cordifolia* leaves *Trichilia monadelpha* leaves	Candidiasis and menstrual pains

*E*	*Alchornea cordifolia* leaves *Vernonia amygdalina* leaves	Gonorrhoea, candidiasis, and all genital infections

STDs, sexually transmitted diseases.

**Table 2:** Measured inhibition zone for herbal preparation C.

Days	1	2	3	Mean ± SD^1^
C1 (mm)	19.0	19.1	19.2	19.1 ± 0.1
C2 (mm)	19.0	19.0	19.1	19.0 ± 0.1
C3 (mm)	19.1	18.9	19.3	19.1 ± 0.2
Mean ± SD^2^	19.0 ± 0.1	19.0 ± 0.1	19.2 ± 0.1	

SD, standard deviation; C1–C3, triplicate discs impregnated with herbal preparation C. The statistical difference was assessed using a two-way ANOVA. ^1^The impregnated discs used did not affect the zone of inhibition (*p*=0.6553), and ^2^the different days did not affect the zone of inhibition (*p*=0.1254).

## Data Availability

The data supporting the current study are given in the article.
